# Co-detection of Pandemic (H1N1) 2009 Virus and Other Respiratory Pathogens

**DOI:** 10.3201/eid1612.091697

**Published:** 2010-12

**Authors:** Kassi Koon, Catherine M. Sanders, Jessica Green, Leslie Malone, Holly White, Delineliz Zayas, Rebecca Miller, Stanley Lu, Jian Han

**Affiliations:** Author affiliations: HudsonAlpha Institute for Biotechnology, Huntsville, Alabama, USA (K. Koon, C.M. Sanders, J. Han);; Diatherix Laboratories, Huntsville (J. Green, L. Malone, H. White, D. Zayas, R. Miller, S. Lu)

**Keywords:** Influenza A, H1N1 subtype, molecular diagnostic testing, respiratory infections, co-detection, viruses, bacteria, dispatch

## Abstract

From May through October 2009, a total of 10,624 clinical samples from 23 US states were screened for multiple respiratory pathogen gene targets. Of 3,110 (29.3%) samples positive for pandemic (H1N1) 2009 virus, 28% contained >1 other pathogen, most commonly *Staphylococcus aureus* (14.7%), *Streptococcus pneumoniae* (10.2%), and *Haemophilus influenzae* (3.5%).

For previous and current influenza A pandemics, postmortem studies have established a strong link between secondary bacterial infections and increased deaths (1,2). Numerous respiratory pathogens can be detected from a single sample by using a multiplex molecular method called target-enriched multiplex PCR (3–6). During the 2006 influenza season, this method was used at Vancouver Children and Women’s Hospital to study 1,742 patients with acute respiratory infections; >2 pathogens were detected for ≈27% of patients studied (7). We used this method to learn more about infections occurring concurrently with pandemic (H1N1) 2009.

## The Study

From May through October 2009, a total of 10,624 clinical samples from 23 states throughout the United States were submitted to Diatherix Laboratories (www.diatherix.com; Huntsville, AL, USA) and screened for multiple respiratory pathogen gene targets. Diatherix, a reference laboratory certified by the Clinical Laboratory Improvement Amendments, provides molecular differential detection services based on target-enriched multiplex PCR technology. The respiratory infections panel detects bacterial and viral pathogens associated with respiratory infections and includes targets for the following: adenovirus (types 3, 4, 7, 21), coxsackievirus, echovirus, human metapneumovirus (types A and B), influenza virus (types A and B), parainfluenza virus (types 1–4), respiratory syncytial virus (types A and B), rhinovirus, *Acinetobacter baumannii, Chlamydophila pneumoniae, Haemophilus influenzae, Klebsiella pneumoniae, Legionella pneumophila, Mycoplasma pneumoniae, Neisseria meningitidis, Pseudomonas aeruginosa, Staphylococcus aureus, Streptococcus pneumoniae,* and *Streptococcus pyogenes* (group A). Additionally, targets specific for pandemic (H1N1) 2009 virus were developed, validated, and approved by the US Food and Drug Administration under Emergency Use Authorization provisions for patient testing.

Of the respiratory specimens shipped by overnight mail from the 23 states, >95% were nasopharyngeal swabs in transfer buffer. High-throughput nucleic acid extraction was performed automatically by using KingFisher 96 instrumentation (Thermo Scientific, Hudson, NH, USA) and MagnetX chemistry (Scigenix, Marietta, GA, USA) according to manufacturers’ specifications. Multiplex PCR amplification and Luminex (Austin, TX, USA) liquid suspension detection methods were based on internally validated protocols. Reactions were amplified by using ABI 9700 thermocyclers (Applied BioSystems, Singapore), and the resulting PCR products were detected by using the LiquiChip 200 Workstation (Luminex) according to previously described protocols (3,6).

Of the 10,624 samples studied, 4,690 (44.1%) were negative for all pathogens detectable with the assay. Among the 7,514 (70.73%) samples negative for pandemic (H1N1) 2009 virus, 3 bacterial pathogens predominated: *S. aureus* (875; 11.65%), *S. pneumoniae* (573; 7.63%), and *H. influenzae* (411; 5.47%) (Table 1). The most common viral pathogens in the pandemic (H1N1) 2009–negative samples were from the family *Picornaviridae*: coxsackie/echovirus (650; 8.65%), and rhinovirus (449; 5.98%) (Table 2).

**Table 1:** Results of screening of clinical samples from 23 US states for pandemic (H1N1) 2009 virus and bacterial respiratory targets, May–October 2009*

No. (%) samples	Bacteria detected, no. (%) samples

***S. aureus***	*P. aeruginosa*	*S. pyogenes*	*N. meningitidis*	***H. influenzae***	*C. pneum*	*K. pneum*	*M. pneum*	***S. pneum***	*A. baumannii*
Pandemic (H1N1) 2009 positive, 3,110 (29.270)	**458 (14.727)**	3 (0.096)	4 (0.129)	7 (0.225)	**110 (3.537)**	1 (0.032)	22 (0.707)	1 (0.032)	**316 (10.161)**	34 (1.093)
Pandemic (H1N1) 2009 negative, 7,514 (70.730)	**875 (11.645)**	28 (0.373)	34 (0.452)	16 (0.213)	**411 (5.470)**	8 (0.106)	44 (0.586)	8 (0.106)	**573 (7.626)**	152 (2.023)
Total, 10,624 (100.000)	**1,333 (12.547)**	31 (0.292)	38 (0.358)	23 (0.216)	**521 (4.904)**	9 (0.085)	66 (0.621)	9 (0.085)	**889 (8.367)**	186 (1.751)

*Screening for *Legionella pneumophilia* detected no bacteria in any samples. *S. aureus, Staphylococcus aureus; P. aeruginosa, Pseudomonas aeruginosa; S. pyogenes, Streptococcus pyogenes; N. meningitidis, Neisseria meningitidis; H. influenzae, Haemophilus influenzae; C. pneum, Chlamydophila pneumoniae; K. pneum, Klebsiella pneumoniae; M. pneum*, *Mycoplasma pneumoniae;* S. *pneum, Streptococcus pneumoniae*; *A.* *baumannii*, *Acinetobacter baumannii*. **Boldface** indicates predominant pathogens.

**Table 2:** Results of screening for of clinical samples from 23 US states for pandemic (H1N1) 2009 virus and respiratory pathogen gene targets, May–October 2009*

No. (%) samples	Viruses detected, no. (%) samples							
	Adeno	Coxackie/echo	Metapneumo	Influenza A	Influenza B	Parainfluenza	RS	Rhino
Pandemic (H1N1) 2009 positive, 3,110 (29.270)	1 (0.032)	13 (0.418)	0	0	0	3 (0.096)	0	7 (0.225)
Pandemic (H1N1) 2009 negative, 7,514 (70.730)	17 (0.226)	**650 (8.651)**	14 (0.186)	3 (0.040)	2 (0.027)	173 (2.302)	3 (0.040)	**449 (5.976)**
Total, 10,624 (100.000)	18 (0.169)	**663 (6.240)**	14 (0.132)	3 (0.028)	2 (0.019)	176 (1.656)	3 0.(028)	**456 (4.292)**

*RS, respiratory syncytial. **Boldface** indicates predominant pathogens.

Of the 10,624 samples studied, 3,110 (29.3%) were positive for pandemic (H1N1) 2009 virus, representing 52.4% of samples positive for any pathogen. Among pandemic (H1N1) 2009 virus–positive samples, >1 other pathogen was co-detected for 28% (Figure). The most commonly co-detected pathogens were *S. aureus* (458; 14.73%), *S. pneumoniae* (316; 10.16%), and *H. influenzae* (110; 3.54%) (Table 1).

**Figure:**
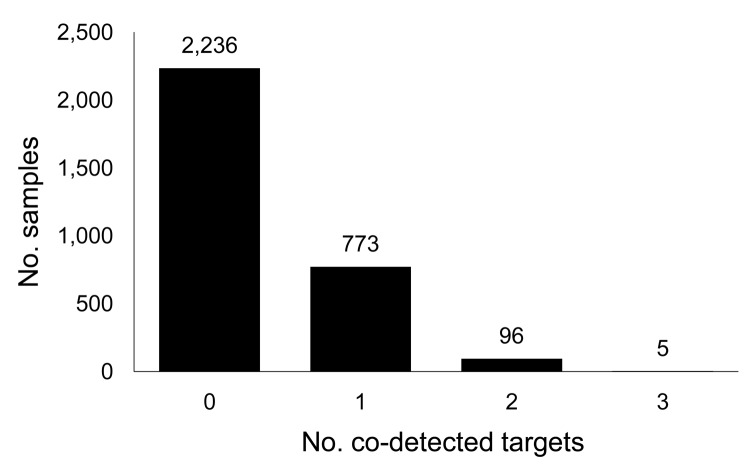
Respiratory pathogens co-detected with pandemic (H1N1) 2009 virus in clinical samples from 23 US states, May–October 2009.

A significant difference (*t* = 25.6, p = 0.01) was found for the age distribution between patients with positive and negative pandemic (H1N1) 2009 virus results. The mean ± SD age was 19.64 (±14.45) years for those who were pandemic (H1N1) 2009 positive and 29.67 (±19.74) years for those who were negative. The median age of the 5 patients for whom 3 other pathogens were co-detected with pandemic (H1N1) 2009 virus (Figure) was 15.5 years. *S. pneumoniae* was detected in all 5 of these samples. For most, the other 2 pathogens were bacteria; for only 1, a virus (parainfluenza) was detected. Of the 96 samples in which pandemic (H1N1) 2009 virus and 2 other pathogens were co-detected (Figure), 30 (31.25%) contained *S. pneumoniae* and *H. influenzae*. The median age of these 30 patients was 4.25 years, whereas the median age of all 96 patients was 8.2 years. The median age for the 28% of patients for whom >1 other target was detected was 11.8 years.

## Conclusions

The main finding of this large-scale clinical study was the co-detection of multiple pathogens with the pandemic influenza virus strain. In 44% of samples, no pathogens were detected, which may represent infection with common pathogens not detected by the assay. For example, bocavirus and all coronavirus groups not detected by the assay account for ≈12% and 5%–10%, respectively (8,9), of respiratory infections. An expanded test menu may improve the detection rate for such pathogens.

This study raises 2 questions. First, does co-detection equal co-infection? Second, and more practical, does co-detection change the clinical outcome? We chose the word co-detection rather than co-infection or co-colonization because co-infection means all identified microorganisms contributed to the pathogenic effect, and co-colonization may not indicate the causative agent. Co-detection indicates that >1 other pathogen was detected in a sample. The differences among the definitions have etiologic meaning, but the data presented here cannot be used directly to address etiology.

Most samples in this study were nasal swabs rather than upper or lower respiratory tract samples. Nasal swab samples have greater potential for contamination with normal flora, particularly *S. aureus.* No data on asymptomatic carriers were available because these persons rarely seek healthcare. However, these findings raise questions about the effectiveness of the single-agent etiology approach toward infectious diseases. Pandemic (H1N1) 2009 virus and multiple other pathogens are often detected during autopsy (1,2), indicating that co-infection may play a major role in the disease process. In addition, detection of multiple pathogens is associated with increased critical illness in children (7).

The Centers for Disease Control and Prevention identified “the need for early recognition of bacterial pneumonia in persons with influenza” (2). However, no suggestions were provided for meeting this need. Furthermore, the Centers “underscore the importance of managing patients with influenza who also might have bacterial pneumonia with both empiric antibacterial therapy and antiviral medications” (2) without identifying measures that would make this task tangible. Current practices of clinical diagnosis based on signs and symptoms inherently lack this type of information.

The true value of a multiplex molecular method of screening for infectious respiratory agents depends on the clinical relevance. Among the samples with >1 positive results, 53% had positive results for viral pathogens without co-detection of bacterial pathogens. For these patients, prescription of antimicrobial drugs on the basis of clinical findings alone could serve to spread drug resistance through selective pressure on normal flora. Furthermore, limited secondary treatment resources, such as oseltamivir administration during a pandemic, could be prioritized on the basis of screening results. Of the 10,624 samples studied, 70.7% were negative for the pandemic (H1N1) 2009 virus strain.

Our findings suggest that multiplex screening for respiratory pathogens is useful for providing rapid surveillance information to inform physicians who would otherwise base decisions on clinical signs and symptoms alone. Electronic reporting of empirical laboratory respiratory pathogen detection provided by a Clinical Laboratory Improvement Amendments–approved laboratory can greatly enhance surveillance data collection (10). Because most states have the authority to collect data of public health relevance (10), the screening service provided by the Diatherix Laboratories could facilitate reporting of notifiable diseases.
